# Study on the Characteristics of Trackside Acoustic Flow Field of High-Speed Train under the Influence of Crosswind

**DOI:** 10.3390/s23208537

**Published:** 2023-10-18

**Authors:** Xing Zhao, Lei Zhang, Lin Li, Qiying Feng

**Affiliations:** College of Locomotive and Rolling Stock Engineering, Dalian Jiaotong University, Dalian 116028, China; zhang2934338430@163.com (L.Z.); 18842626868@163.com (L.L.); 18103536506@163.com (Q.F.)

**Keywords:** trackside acoustic detector system, crosswind, acoustic streaming field distribution

## Abstract

During the on-track acoustic detection process, a potential flow model and an acoustic finite element mathematical model based on synthetic wind are utilized, taking into account the combined effects of vehicle speed, wind direction angle, and crosswind speed. Simulation and modeling are achieved using Automatic Matching of Acoustic Radiation Boundary Conditions (AML) technology, enabling obtaining a distribution map and sound pressure frequency response curve of the trackside acoustic field under crosswind conditions by setting up field point grids. It is found that sound pressure values at the same location gradually increase as the vehicle speed increases in the frequency range of 10 Hz to 70 Hz, at different vehicle speeds. The sound pressure values and distribution area of the trackside acoustic field are the largest when the crosswind speed is 10 m/s (wind force at level five), allowing for easier location of the sound source when a fault occurs. The study also reveals that under different wind direction angles, the same location’s sound pressure value on the trackside gradually decreases as the wind direction angle increases, to lower than that of the non-crosswind condition, severely hindering the reception and diagnosis of acoustic signals.

## 1. Introduction

The rapid development of China’s high-speed railways has brought a continuous increase in the speed and number of trains running. Although high-speed trains adopt advanced technologies and materials, the bearings of the trains are prone to failures because they are subjected to alternating forces for a long time [1]. Because of this, strengthening the condition monitoring of train bearings so as to improve the safety level of train operation has highly concerned the state and the people. Traditional bearing detection methods are mainly through manual inspection and vibration detection, but there are problems such as low efficiency and poor accuracy. A Trackside Acoustic Detector System (TADS) effectively realizes the early warning of bearing failure by arranging sensor arrays and acquisition and processing systems on both sides of the track [2], providing a strong guarantee of the safety of running trains.

Around the 1980s, the U.S. railroad transportation department began research and exploration on train bearing acoustic detection and diagnostic technology, and formally developed a trackside acoustic diagnostic system. Subsequently, in the 1990s, the sensing device was further optimized, and a microphone array system was adopted as the current practical trackside acoustic detection system. At the same time, also from the 1990s and after a long period of exploration and a large number of repeated tests, the Australian company VIPAC in 1998 successfully developed its Railway Acoustic Monitoring System (Rail BAM), a bearing acoustic diagnostic system [3].

Railroads, roads and airports cause major environmental impacts when in contact with populated areas. With the growth of cities, society is increasingly questioning these problems, and people living in the proximity of railway tracks consider noise the most serious environmental problem [4]. The railway infrastructure in a city is not composed only by the rail network, but also includes depots, stations, washes, and marshalling yards, as well as metro or tram facilities (Vogiatzis and Kouroussis, 2015). The marshalling yards are the areas where the freight trains are decoupled and coupled, becoming a source of screeching rail brakes, peak noise from coupling vehicles, starting diesel engines, and steady noise from locomotive engines and auxiliary systems [5]. Railway transport, both of passengers and freight, is increasing, and new railway lines are planned for environmental reasons. The combination of more frequent railway traffic and faster and heavier trains will, most probably, lead to more disturbances in the near future. The number of trains per se and the presence of ground-borne vibrations induced by railway traffic, and not just the noise level, are of relevance for the perceived annoyance of railway noise [6]. Furthermore, railway noise is often related to vibrations, which induce other negative effects on sleep [7]. Railway noise has generally been considered as the least annoying transportation source, and, therefore, it has not drawn the same attention as road traffic and airport noise. However, recent studies suggest that nocturnal railway noise is as disturbing to sleep as road traffic noise and that railway noise has an important short-term impact on the cardiovascular system and might increase the risk for hypertension [8].

In recent years, the research on trackside acoustic detection systems has, on the one hand, mainly focused on acoustic signal acquisition and analysis technology, track defect detection algorithms, system integration and optimization. In terms of acoustic signal acquisition and analysis techniques, the research on noise elimination methods is an important research direction. For example, [9] proposes an improved dual-microphone active noise cancellation method that can reduce noise more effectively. A new algorithm is also proposed to detect and separate speech signals from noise signals. Ref. [10] proposed a parameterization-based Doppler adaptive correction algorithm, which takes the field effect into account, reduces the error due to the motion of the vehicle body and environmental interference, and improves the accuracy and reliability of train detection. However, the algorithm is not applicable to all scenarios. On the other hand, research on bearing fault diagnosis is also a popular field. And Li Jingjiao [11] proposed a method based on wavelet packet transform and blind source separation, which can extract the fault characteristics of bearing acoustic signals from strong background noise. However, the method requires high computational resources and time.

When the train runs at high speed in a strong wind environment, the flow field around the train becomes exceptionally complex [12], which not only affects the propagation of sound waves in the air, but also increases the aerodynamic force and aerodynamic moment to which the train is subjected, and at this time, the train may be derailed at any time, overturned and placed at risk of other serious accidents [13].

Domestic and foreign research on the impact of wind on the operational safety of high-speed trains is currently focused on the field of aerodynamics in strong wind environments and the field of aerodynamic noise from ultra-high-speed trains. Xie Hongtai [14] used numerical simulation methods to study the aerodynamic characteristics of a certain type of high-speed train under the coupling effect of train wind and gale, which provides theoretical guidance and technical support for China’s independent research and development of high-performance trains. Hao Nansong [15] carried out wind tunnel experiments for a certain type of rolling stock and found that there is low- and medium-frequency broadband noise in the bogie region, which provides strong support for the aerodynamic noise simulation optimization technology of high-speed trains [16,17,18,19,20,21]. Jiao et al. [22] found that a strong dipole sound source exists in the bogie region of a certain type of rolling stock through a large vortex simulation study. Zhang Yadong et al. [23] used wind tunnel experiments to analyze the noise contribution and characteristics of high-speed trains and identified the main noise sources of high-speed trains as well as the magnitude of the aerodynamic noise contributions from the pantographs and various parts of the bogies. All of these studies provide strong support for the further development of future high-speed train technology, which is of great significance for future high-speed train design and operation.

Side wind, as an important external factor in high-speed train operation, will have a great impact on the train and its surroundings, and the study of the trackside acoustic flow field of high-speed trains under the influence of sidewind can improve the safety of train operation. Because the whole-field motion of the fluid affects the propagation of acoustic waves in the medium, resulting in errors in the signals received by the sensor array, it can cause miscalculation of faults or failure to realize early warnings, thus threatening the safety of train operation [24]. In order to solve this problem, this paper places the vibration source in the position of the bearing cover, where the train bearings are in direct contact with the outside world, introduces the sidewind flow field in the process of train driving, and analyzes the characteristics of the trackside acoustic flow field of a high-speed train under the influence of the sidewind by changing the parameters of the vehicle speed, wind speed, and wind direction angle, which provides a value for the subsequent design of the train trackside acoustic detection system and the research on fault diagnosis. In addition, the study of the trackside acoustic flow field under the influence of crosswinds can provide an in-depth understanding of the noise distribution and its changes when the train is running, so as to optimize the design of the train, reduce the noise, improve the comfort of the train, and reduce the environmental pollution.

## 2. Calculation Model for High-Speed Trains

### 2.1. Train Model Simplification

Based on the CR400AF model of the Fuxing high-speed train, which was officially put into operation in 2017, the high-speed train model is simplified as follows, with a reduced computational volume and satisfying requirements for relevant accuracy:

(1) Reduce the number of train compartments and the length-to-slender ratio of the train. This paper adopts the whole train calculation model of three formations, i.e., head car, middle car and tail car, in which the head car and tail car are identical in shape, the length is 27 m, the length of the middle car is 25 m, the width of the car is 3.36 m, and the height of the car is 4.05 m.
(2) Simplify the design of the car body shape. Ignore complex devices such as the pantograph above the roof, the structure connecting the carriages, the windows and the doors, and build a smooth body model to reduce the computational complexity.
(3) Simplify the bogie of the car body. The bogie consists of five parts: frame, travel system, suspension system, braking system, and traction device. According to the focus of this study, only the bogie bearings, frame and wheel structure need to be retained; the bogie wheelbase is 2.5 m, and the wheel diameter is 0.95 m.

**Figure 1:**
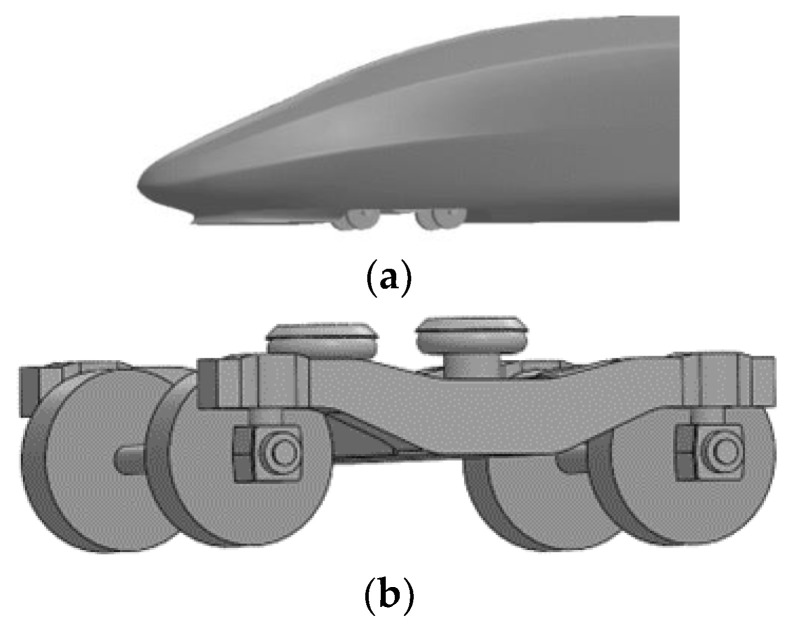

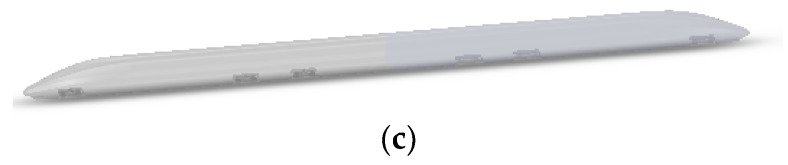
Simplified train model diagram. (**a**) Head and tail; (**b**) Bogie; (**c**) Overall view.

The simplified structural model size table of the rolling stock and the train model diagram are shown in Figure 1.

### 2.2. Selection of the Flow Field Region

**Figure 2:**
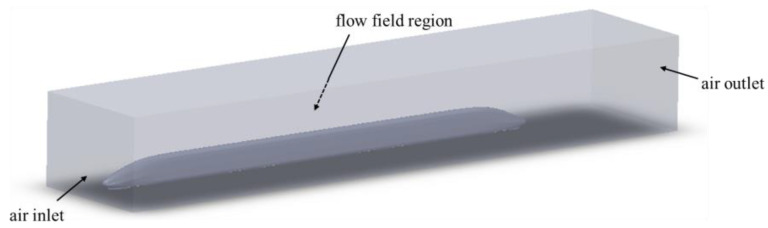
Schematic diagram of acoustic field simulation model.

According to the analysis of the actual situation, it is known that the flow field region is infinite when the high-speed train operates in the sidewind environment. However, some scholars have proved that the realism of the simulation results of the computational domain is too large to change significantly. Therefore, the infinitely large computational domain is truncated into a finite flow field region without affecting the flow field around the train. During the driving process of the train, transverse flow and longitudinal vortex are generated due to the uneven distribution of airflow in the tail car. Therefore, in order to simulate the real flow field situation, the flow field boundary is placed close to the head car and far from the tail car [25]. In addition, in order to simulate the real train bottom structure, it is necessary to keep a certain distance between the bottom surface of the train model and the ground. According to previous studies, the length from the rear of the train to the flow field boundary is 5–10 times the height of the train itself, which can make the fluid distributed evenly after passing the rear of the train. Therefore, the size of the computational domain is 120 m long, 40 m wide, and 25 m high. The specific model is shown in Figure 2.

### 2.3. Model Meshing

Since the head surface of the train model used in this paper is irregular and the bogie structure is complex, it is a complex irregular model. Compared with the structural grid, the unstructured grid can better discretize the model. Therefore, the unstructured grid is chosen to delineate the area between the train and the flow field area in this paper. To ensure the accuracy of acoustic simulation calculation, at least six cells need to be divided within each minimum wavelength. In the linear cell simulation, the speed of sound c and the maximum calculated frequency $f_{\max}$ are known, and cell length should be adjusted by Equation (1) to meet the maximum frequency range that can be covered.

(1) $$L\leq \frac{c}{6f_{\max}}$$

**Figure 3:**
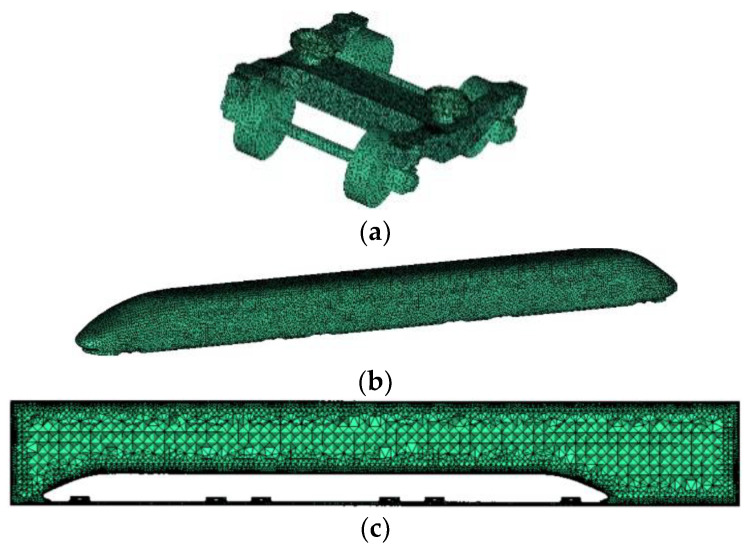
Grid partitioning model diagram. (**a**) Bogie grid; (**b**) Body grid; (**c**) Grid of flow field areas.

The maximum cell size of the bogie grid is set to 60 mm, the grid at the bogie is encrypted to improve the grid quality and capture the details of the bogie, and the maximum cell grid size of the moving train surface is set to 280 mm to reduce the calculation volume and calculation time. After the model is meshed, the total number of meshes is about 6.8 million. The high-speed train model is meshed as shown in Figure 3.

### 2.4. Acoustic Flow Field Simulation Process

**Figure 4:**
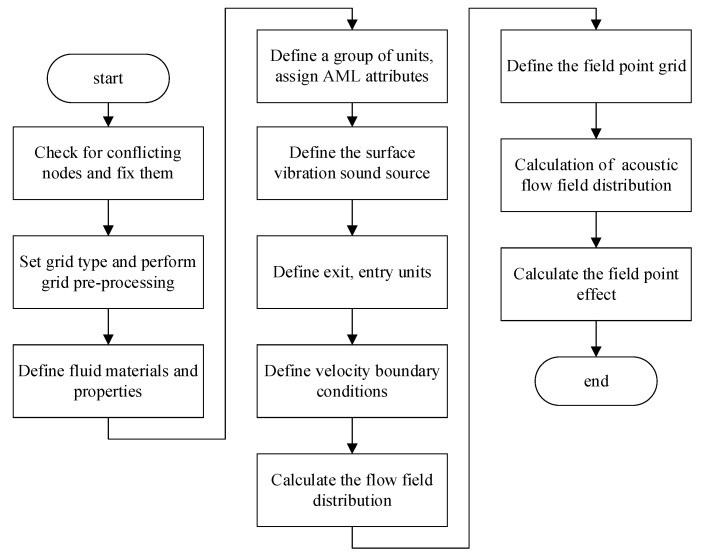
Flowchart of the acoustic simulation process.

The finite element analysis method using the acoustic module of LMS Virtual. Lab (13.6) software is applied to simulate the trackside sound field of high-speed trains. The specific flow chart is shown in Figure 4.

### 2.5. Sound Source Location and Field Point Grid Definition

**Figure 5:**
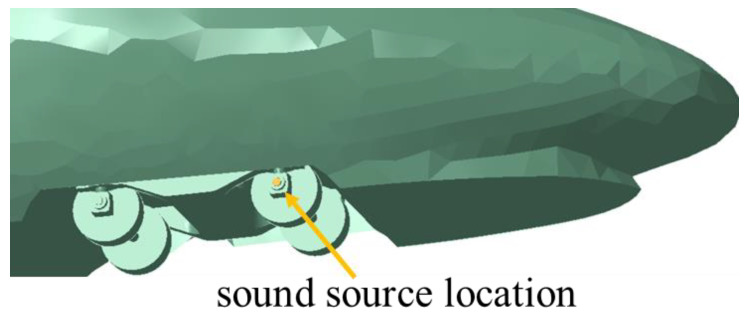
Diagram of the location of the surface vibration sound source.

**Figure 6:**
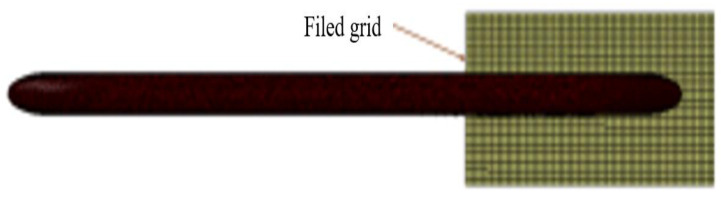
Field point grid diagram.

(1) Surface vibration sound source. Since the high-speed train trackside sound field is aimed at the sound waves from bearing faults, and the sound from bearing faults is finally propagated to the outside world through the bearing cover, the surface vibration sound source is placed at the axle box cover position, which can more accurately simulate the propagation of sound waves when bearing faults are generated. The placement of the sound source in this paper is shown in Figure 5.

(2) Field point size. The field point grid is too large to affect the calculation time and calculation accuracy, so it should be set appropriately. The size and location of the field point grid used in this paper are shown as the yellow grid in Figure 6.

## 3. High-Speed Train Trackside Sound Field Distribution Map Analysis

### 3.1. Mathematical Calculation Modeling under the Joint Influence of Vehicle Speed, Wind Direction Angle and Sidewind Speed

**Figure 7:**
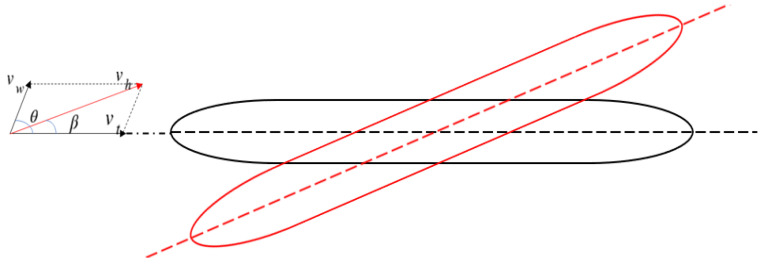
Schematic diagram indicating the direction of crosswinds for high-speed trains.

In the study of the sidewind problem, the coupling between the train wind and the ambient wind needs to be analyzed. To achieve this goal, the flow field continuation method is used. The basic idea of the method is to achieve the desired effect by adjusting the angle of the train. The specific model is shown in Figure 7.

In Figure 7, $v_{t}$ represents the train wind speed; $v_{w}$ represents the ambient wind speed; $v_{h}$ represents the coupled wind speed of train wind and sidewind; θ represents the wind angle; β represents the side deflection angle. In addition, the black area represents the direction of the train, and the red area represents the direction of the crosswind. In calculating the effect of sidewind on train operation, the coupled wind speed of train wind and sidewind speed $v_{h}$ is used for simulation. These quantities follow the specific relations of (2) and (3):

(2) $$v_{h}=\sqrt{v_{t}{}^{2}+2v_{t}v_{\omega }\cos\theta +v_{\omega }{}^{2}}$$

(3) $$\beta =\arccos\frac{v_{t}{}^{2}+v_{h}{}^{2}+v_{\omega }{}^{2}}{2v_{t}v_{h}}$$

### 3.2. Analysis of the Effect of Different Vehicle Speeds on the Sound Field Calculation

In order to study the variation in the trackside sound field at different driving speeds of high-speed trains, simulations were performed to analyze the distribution characteristics of the trackside sound field when the train is running at 30 km/h, 60 km/h, 90 km/h, and 120 km/h with 6 m/s level 4 wind and 20° wind direction angle. Table 1 shows the specific values of each simulation parameter.

**Figure 8:**
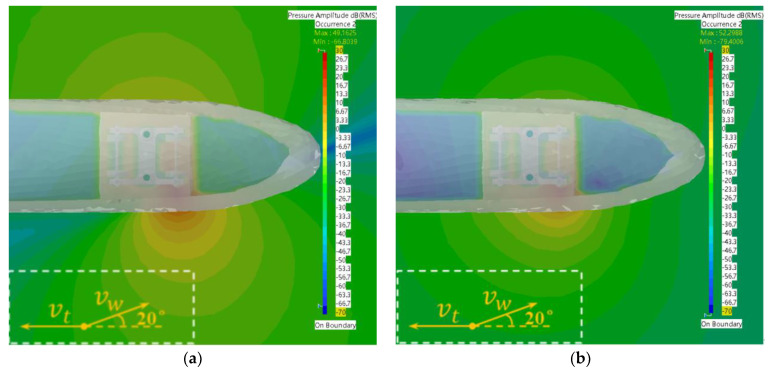

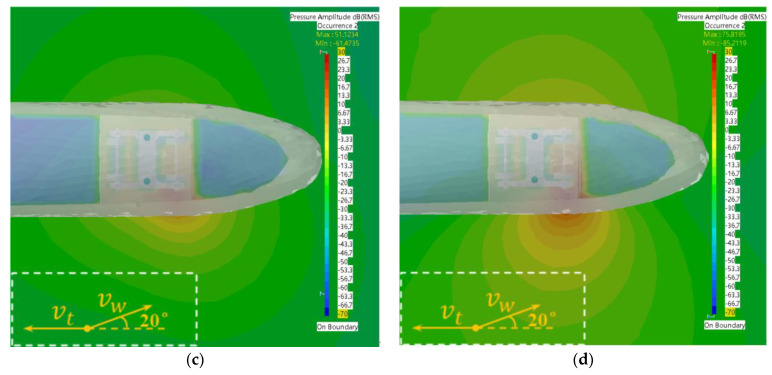
Sound field distribution maps at different vehicle speeds under a frequency of 30 Hz. (**a**) Vehicle speed of 30 km/h; (**b**) Vehicle speed of 60 km/h; (**c**) Vehicle speed of 90 km/h; (**d**) Vehicle speed of 120 km/h.

According to the simulation results in Figure 8, it can be found that the shape of the trackside sound field is roughly circular, radiating outward, but there are large differences in the propagation direction of the sound waves at different vehicle speeds. This is mainly due to the difference in airflow distribution around the vehicle, which leads to different resistance and scattering of sound waves in the process of propagation. The propagation direction of sound waves on the windward side varies significantly, while the propagation direction of sound waves on the leeward side is relatively stable. In addition, the change in sound field distribution is related to the propagation characteristics of sound waves in a fluid medium: when the sound waves encounter obstruction, they will produce scattering, reflection or refraction and other phenomena, and these phenomena will also lead to changes in the sound field distribution. At different vehicle speeds, the lateral propagation of sound waves near the sound source is more desirable. In addition, at this frequency, the sound pressure value near the fault source is significantly higher than at other locations. The sensor at this time can receive an effective signal for accurate localization of the fault source.

### 3.3. Analysis of the Effect of Different Wind Angles on the Calculation of the Sound Field

In order to investigate the effect of wind angle on the distribution of the trackside sound field, the distributions of the sound field with wind angles of 10°, 30°, 50° and 70° at a travel speed of 110 km/h and wind speed of 7 m/s were considered. The corresponding parameter values are given in Table 2.

**Figure 9:**
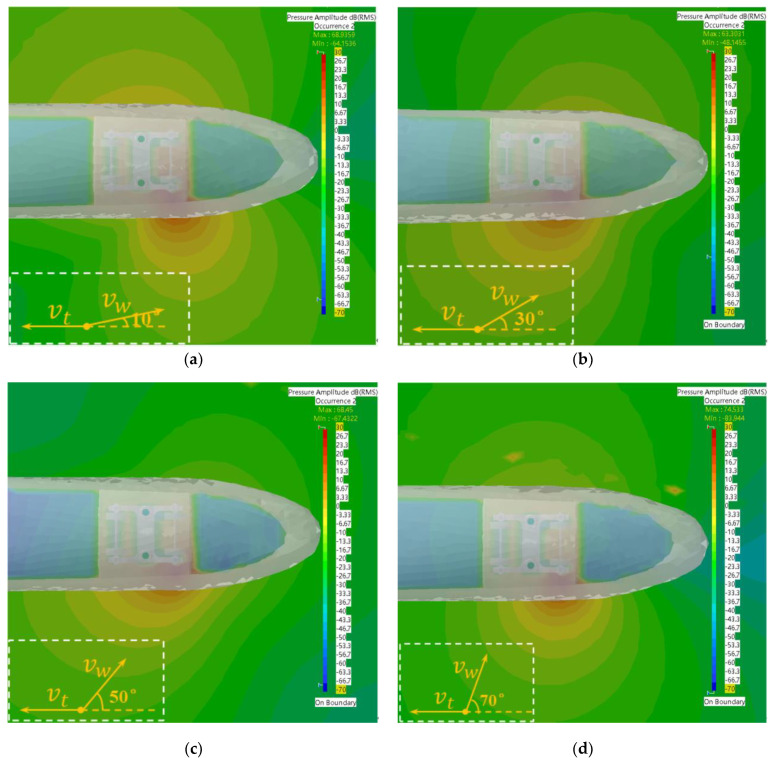
Sound field distribution maps at different wind direction angles under a frequency of 30 Hz. (**a**) Wind direction angle of 10°; (**b**) Wind direction angle of 30°; (**c**) Wind direction angle of 50°; (**d**) Wind direction angle of 70°.

According to the simulation results in Figure 9, it can be found that the boundary contour of the sound field distribution at this frequency was uniform and neat; however, the propagation direction of the sound field and the gradient of the sound pressure varied with the wind angle, and the sound field distribution on both sides of the car body did not have symmetry. Specifically, at a wind angle of 10°, the propagation direction of sound waves on the windward side tilted toward the opposite direction of train travel, while at other wind angles, it shifted toward the direction of train travel. In particular, the wind angle of 50° had the largest offset angle. As the propagation distance increased, the sound pressure of the trackside sound field at a wind angle of 10° changed more slowly than at the other angles. Overall, the longitudinal propagation of sound waves was more desirable at the four wind angles. In addition, the sound pressure values on the windward side at this frequency were generally higher than those on the leeward side, so the acoustic sensor array could capture the signals more easily and thus locate the bogie position of the noise source more effectively.

### 3.4. Analysis of the Effect of Different Side Wind Speeds on the Sound Field Calculation

A high-speed train traveling at 120 km/h was used as the research object, and the distribution characteristics of the sound field at the trackside of the high-speed train were simulated and analyzed under the wind angle of 20° for wind speeds of 8 m/s, 10 m/s, 12 m/s and 14 m/s. The specific values of the simulation parameters are shown in Table 3.

**Figure 10:**
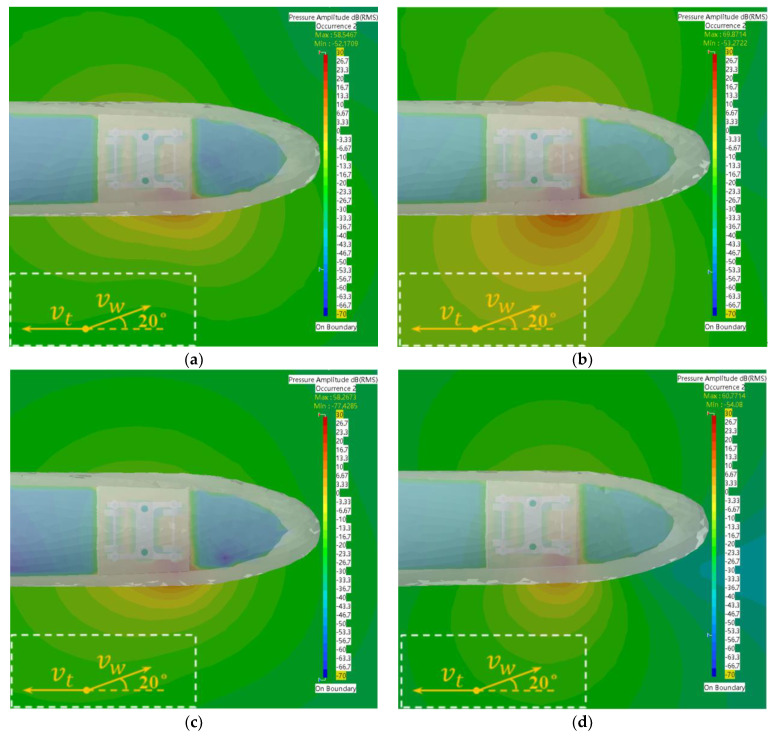
Sound field distribution maps at different wind speeds under a frequency of 30 Hz. (**a**) Side wind speed of 8 m/s; (**b**) Side wind speed of 10 m/s; (**c**) Side wind speed of 12 m/s; (**d**) Side wind speed of 14 m/s.

According to the simulation results in Figure 10, it can be found that the sound wave propagation range, direction and sound pressure values varied at different sidewind speeds at 30 Hz. When the sidewind speed was 8 m/s and 12 m/s, it was easier for the acoustic wave to propagate laterally, resulting in a clear horizontal and vertical contrast in the sound field along the lateral distribution. However, when the sidewind speed was 10 m/s and 14 m/s, the sidewind was more favorable for longitudinal propagation as the distance of acoustic wave propagation outward increased. Overall, the propagation direction of the windward side of the trackside sound field at all wind speeds except for the sidewind speed of 8 m/s was shifted toward the direction of train travel. At this frequency, the sound pressure value at the source of the fault was significantly different from that of other regions, which was favorable for identification of the fault signal. The largest difference was found at a sidewind speed of 10 m/s, while the smallest difference was found at a sidewind speed of 14 m/s. Determining the appropriate detection method at different sidewind speeds is essential for accurate identification of the fault signal.

## 4. High-Speed Train Trackside Sound Pressure Frequency Response Graph Analysis

To investigate in depth the effect of vehicle speed and frequency on the distribution of the trackside sound field, the sound pressure levels at different frequencies were recorded by using a microphone at the location of the detection point, and then the data were processed to generate a sound pressure frequency response map to assess the acoustic characteristics of the location.

**Figure 11:**
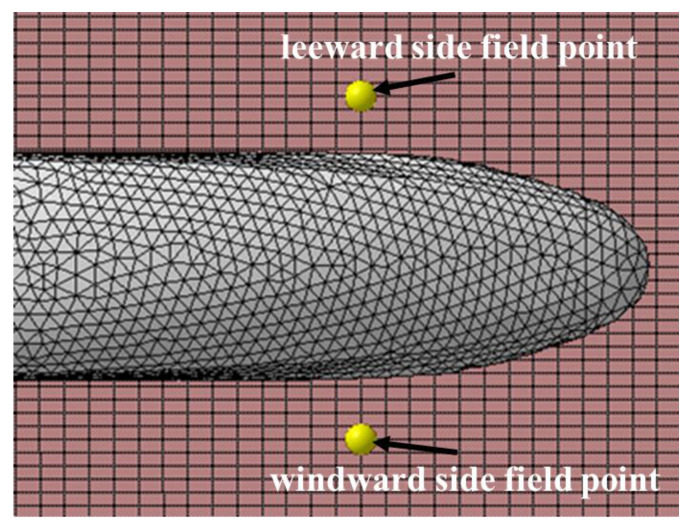
Sound field distribution chart.

The analysis of the results could provide basic data support for further acoustic design and optimization. The locations of the field points on both sides of the high-speed train in the following study are shown in Figure 11.

### 4.1. Influence of Vehicle Speed

**Figure 12:**
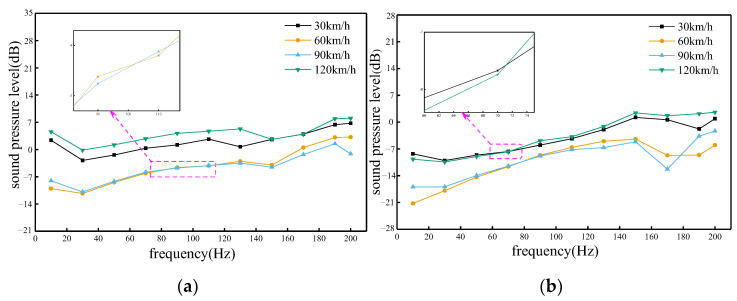
Sound pressure level response graphs at various frequencies and vehicle speeds. (**a**) Windward side; (**b**) Leeward side.

According to Figure 12, it can be concluded that the trend of the effect on the sound pressure values at different vehicle speeds and frequency ranges does not have a monotonic relationship. In the frequency range of roughly 82 Hz to 110 Hz on the windward side, the speed variation had a more significant effect on the sound pressure value. Before this frequency range, high-speed trains produced stronger excitation on the track, which caused more intense vibration, resulting in more sound waves being generated and propagated around the track, so the sound pressure value increased with the increase in speed. On the leeward side, the sound pressure value was most severely affected when the speed was 90 km/h. However, the sound pressure value was always the highest when the train was running at 120 km/h. This is because the faster the train runs in the wind environment on the same side, the farther the sound waves travel per unit time, and the greater the sound pressure value that will be generated at the same time.

### 4.2. Influence of Wind Direction Angle

**Figure 13:**
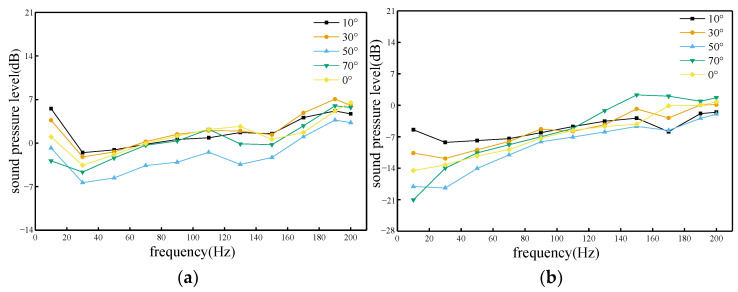
Sound pressure level response graphs at various frequencies and wind direction angles. (**a**) Windward side; (**b**) Leeward side.

According to Figure 13, it can be concluded that the distribution characteristics of the trackside sound field of high-speed trains on the windward and leeward sides are different. The sound pressure values of the trackside sound field on the leeward side of the train are smaller than those on the windward side, indicating that the sound waves are not compressed during the propagation process, and the sound waves are relatively sparse. At different frequencies, the sound wave propagation was most severely attenuated when the wind angle was 50°. On the windward side, the frequency range was from 10 Hz to 70 Hz, and the sound pressure values were higher at wind direction angles of 10° and 30°, while the wind direction angle was larger at 50° and 70°, which hindered the propagation of sound waves. On the leeward side, the frequency range was 50 Hz~150 Hz, and the sound pressure values were higher at all wind angles except for the wind direction angle of 50°.

### 4.3. Influence of Side Wind Speed

**Figure 14:**
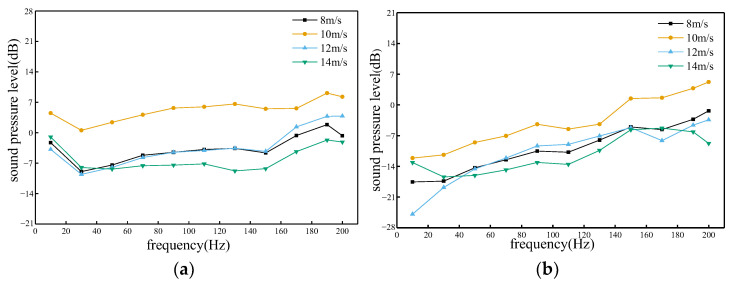
Sound pressure level response graphs at various frequencies and wind speeds. (**a**) Windward side; (**b**) Leeward side.

According to Figure 14, it can be concluded that at different frequencies, both on the windward and leeward sides, the sound pressure value at a sidewind speed of 10 m/s is the largest, which means that this sidewind speed is the most favorable for the propagation of sound waves. When the frequency range of the windward side was about 50 Hz to 200 Hz, the sound pressure value was the smallest when the sidewind speed was 14 m/s. On the other hand, when the frequency range of the leeward side was between about 45 Hz and 150 Hz, the sound pressure value was the smallest when the sidewind speed was 14 m/s. This indicates that a sidewind speed of 14 m/s is most unfavorable for sound wave propagation at frequencies of 50 Hz to 200 Hz on the windward side and 45 Hz to 150 Hz on the leeward side.

## 5. Conclusions

(1) Sidewinds can have an effect on the sound field distribution at the trackside. When a sidewind exists, the fluid velocity on the train surface increases at the same speed, and the train surface is enhanced by the gas flow.
(2) At different vehicle speeds, the acoustic pressure value at the same location increases gradually with the increase in vehicle speed in the frequency range of 10 Hz to 70 Hz. And when the vehicle speed is 120 km/h, the degree of change in the acoustic signal is not obvious, so it is easier to detect and capture if a bearing failure occurs.
(3) Under different wind angles, as the wind angle increases, the sound pressure value at the same position of the trackside sound field gradually becomes smaller and lower than that of the non-sidewind situation. When the frequency range is between 10 Hz~150 Hz, the smaller the wind angle, the more favorable for location of the fault sound source.
(4) At different sidewind speeds, the sound pressure value and distribution area of the train trackside sound field are the largest when the sidewind speed is 10 m/s (force 5 wind), which is very favorable for the reception and diagnosis of acoustic signals.

## Figures and Tables

**Table 1:** The parameter calculation table for different train speeds.

Vehicle Speed $v_{t}$ (km/h)	Slip Angle β (°)	Coupling Wind Speed $v_{h}$ (m/s)
$v_{t1}=30$	$\beta_{1}=8.4$	$v_{h1}=14.12$
$v_{t2}=60$	$\beta_{2}=5.2$	$v_{h2}=22.40$
$v_{t3}=90$	$\beta_{3}=3.8$	$v_{h3}=30.71$
$v_{t4}=120$	$\beta_{4}=3.0$	$v_{h4}=39.03$

**Table 2:** The parameter calculation table for different wind angels.

Wind Direction Angle θ (°)	Slip Angle β (°)	Coupling Wind Speed $v_{h}$ (m/s)
$\theta_{1}=10$	$\beta_{1}=2.4$	$v_{h1}=37.48$
$\theta_{2}=30$	$\beta_{2}=5.4$	$v_{h2}=36.78$
$\theta_{3}=50$	$\beta_{3}=8.7$	$v_{h3}=35.46$
$\theta_{4}=70$	$\beta_{4}=11.4$	$v_{h4}=33.62$

**Table 3:** The parameter calculation table for different wind speeds.

Wind Speed $v_{w}$ (m/s)	Slip Angle β (°)	Coupling Wind Speed $v_{h}$ (m/s)
$v_{w}=8$	$\beta_{1}=3.8$	$v_{h1}=40.94$
$v_{w}=10$	$\beta_{2}=4.6$	$v_{h2}=42.87$
$v_{w}=12$	$\beta_{3}=5.2$	$v_{h3}=44.80$
$v_{w}=14$	$\beta_{4}=5.9$	$v_{h4}=46.74$

## Data Availability

The data presented in this study are available upon request from the corresponding author.
